# Early management of patients with aneurysmal subarachnoid hemorrhage in a hospital with neurosurgical/neuroendovascular facilities: a consensus and clinical recommendations of the Italian Society of Anesthesia and Intensive Care (SIAARTI)–Part 1

**DOI:** 10.1186/s44158-022-00042-x

**Published:** 2022-03-31

**Authors:** Edoardo Picetti, Andrea Barbanera, Claudio Bernucci, Alessandro Bertuccio, Federico Bilotta, Edoardo Pietro Boccardi, Tullio Cafiero, Anselmo Caricato, Carlo Alberto Castioni, Marco Cenzato, Arturo Chieregato, Giuseppe Citerio, Paolo Gritti, Luigi Lanterna, Roberto Menozzi, Marina Munari, Pietro Panni, Sandra Rossi, Nino Stocchetti, Carmelo Sturiale, Tommaso Zoerle, Gianluigi Zona, Frank Rasulo, Chiara Robba

**Affiliations:** 1 Department of Anesthesia and Intensive Care, Azienda Ospedaliero-Universitaria di Parma, Parma, Italy; 2 Department of Neurosurgery, “SS Antonio e Biagio e Cesare Arrigo” Hospital, Alessandria, Italy; 3 Department of Neuroscience and Surgery of the Nervous System, ASST Papa Giovanni XXIII Hospital, Bergamo, Italy; 4 Department of Anesthesiology and Critical Care, Policlinico Umberto I Hospital, La Sapienza University of Rome, Rome, Italy; 5 Department of Interventional Neuroradiology, ASST Grande Ospedale Metropolitano Niguarda, Milan, Italy; 6 Department of Anesthesia and Intensive Care Unit, AORN Cardarelli, Naples, Italy; 7 Department of Anesthesia and Critical Care, IRCCS A. Gemelli University Polyclinic Foundation, Rome, Italy; 8 Department of Anesthesia and Intensive Care, IRCCS Institute of Neurological Sciences of Bologna, Bologna, Italy; 9 Department of Neurosurgery, ASST Grande Ospedale Metropolitano Niguarda, Milan, Italy; 10 Neurointensive Care Unit, Department of Neuroscience and Department of Anesthesiology, ASST Grande Ospedale Metropolitano Niguarda, Milan, Italy; 11 School of Medicine and Surgery, University Milano-Bicocca, Milan, Italy; 12 Department of Anesthesia and Critical Care Medicine, Papa Giovanni XXIII Hospital, Bergamo, Italy; 13 Interventional Neuroradiology Unit, University Hospital of Parma, Parma, Italy; 14 Anesthesia and Intensive Care, Padua University Hospital, Padua, Italy; 15 Department of Neuroradiology, San Raffaele Hospital, Milan, Italy; 16 Neuroscience Intensive Care Unit, Department of Anesthesia and Critical Care, Fondazione IRCCS Ca’ Granda Ospedale Maggiore Policlinico, Milan, Italy; 17 Department of Pathophysiology and Transplantation, University of Milan, Milan, Italy; 18 Neurosurgery Unit, IRCCS Istituto delle Scienze Neurologiche Ospedale Bellaria di Bologna, Bologna, Italy; 19 Department of Neurosurgery, Policlinico San Martino Hospital, IRCCS for Oncology and Neuroscience, Genoa, Italy; 20 Department of Anesthesia, Intensive Care and Emergency Medicine, Spedali Civili University Hospital, Brescia, Italy; 21 Anesthesia and Intensive Care, San Martino Policlinico Hospital, IRCCS for Oncology and Neurosciences, Genoa, Italy; 22 Department of Surgical Sciences and Integrated Diagnostics, University of Genoa, Genoa, Italy

**Keywords:** Subarachnoid hemorrhage, Hemodynamic management, Surgical management, Blood pressure, Aneurysm treatment

## Abstract

**Background:**

Issues remain on the optimal management of subarachnoid hemorrhage (SAH) patients once they are admitted to the referring center, before and after the aneurysm treatment. To address these issues, we created a consensus of experts endorsed by the Italian Society of Anesthesia and Intensive Care (SIAARTI). In this manuscript, we aim to provide a list of experts’ recommendations regarding the early management of SAH patients from hospital admission, in a center with neurosurgical/neuro-endovascular facilities, until securing of the bleeding aneurysm.

**Methods:**

A multidisciplinary consensus panel composed of 24 physicians selected for their established clinical and scientific expertise in the acute management of SAH patients with different background (anesthesia/intensive care, neurosurgery, and interventional neuroradiology) was created. A modified Delphi approach was adopted.

**Results:**

Among 19 statements discussed. The consensus was reached on 18 strong recommendations. In one case, consensus could not be agreed upon and no recommendation was provided.

**Conclusions:**

This consensus provides practical recommendations for the management of SAH patients in hospitals with neurosurgical/neuroendovascular facilities until aneurysm securing. It is intended to support clinician’s decision-making and not to mandate a standard of practice.

## Background

Aneurysmal subarachnoid hemorrhage (SAH) represents an important cause of mortality and morbidity, with half of the survivors experiencing persistent neurological deficits [1, 2]. The prognosis of SAH patients depends on multiple non-modifiable factors (as patients’ pre-injury characteristics, the extent of primary cerebral damage, etc.) and on therapeutic interventions [1–3]. The primary goal of the treatment of aneurysmal SAH is the exclusion of the ruptured aneurysm, as rebleeding importantly increases the risk of mortality and poor clinical outcome [1, 2, 4, 5]. Recently, we developed a consensus with clinical recommendations of the Italian Society of Anesthesia and Intensive Care (SIAARTI) on the early management of patients with aneurysmal subarachnoid hemorrhage in a hospital without neurosurgical/neuro-endovascular facilities, and we identified a total of 13 recommendations to provide clinicians with a pragmatic approach in such clinical situations [6]. However, questions remain on the optimal management of SAH patients once they are admitted to the referring center, before and after the aneurysm treatment. Large, multicenter, randomized trial data confirming the effectiveness of treatments are lacking for many of the interventions applied even in this phase, and currently, only a few aspects of SAH management are supported by high-quality evidence [7, 8].

Therefore, we created a consensus of experts endorsed by the SIAARTI regarding the management of SAH patients after admission to the hospital with neurosurgical/neuroradiological facilities, and for clarity, we decided to split the recommendations and discussion considering the clinical approach before and after aneurysm treatment. Specifically, in this manuscript (part 1), we aim to provide a list of experts’ recommendations regarding the early management of SAH patients from hospital admission, in a center with neurosurgical/neuro-endovascular facilities, until the aneurysm treatment.

## Methods

The steering committee (EP, CR, FR) selected a multidisciplinary panel of experts according to their established clinical and scientific expertise in the management of SAH, including a methodologist, neurointensivists, neuroanesthesiologists, neurosurgeons, and neuroradiologists. Two experts were identified as an advisory committee for their clinical and scientific expertise in the field (NS, GC). The Executive Committee of the SIAARTI commissioned the project and supervised the methodology and structure of the consensus. The Steering Committee conceived the project, defined the aims, the timeline and the methodology (engaged with the research group of the SIAARTI for the development of the consensus), set the agenda for meetings and Delphi rounds, and ensured communications with the panel. The steering committee met weekly via teleconferences, from August 2021 to February 2022, using the Zoom platform (Zoom Video Communications), and had 2 meetings with the Research Committee of SIAARTI for the definition of the methodology and an assessment of the on-going work.

### Delphi process

The group conducted a non-systematic review regarding the clinical management of SAH patients admitted to a hospital with neurosurgical/neuro-endovascular facilities, until the aneurysm treatment and afterwards the panel and the steering committee identified the domains and generated a list of questions to be addressed by the panel. An initial list of statements was distributed to the experts for discussion and refinement. According to the panel’s feedback, a set of questions was generated. We used an online tool to conduct the modified iterative Delphi process [9, 10]. Using three online surveys—distributed from September 2021 to January 2022—the Consensus panel members were asked to express their degree of agreement and voted independently, with the possibility to add specific comments during the first two voting rounds. Comments were used to further refine the questions. Finally we proposed to vote on practical statements (recommendations) based on the answer to the questions. Three thresholds were pre-defined: when more than 85% of the panelist agreed on a specific statement, we issued a *strong recommendation*, while a *weak recommendation* could be possible in case of agreement by 75–85% of the panelists*.* If less than 75% of the participant endorsed a statement, no recommendation could be issued*.*

After each round, we analyzed answers to spot heterogeneity and inconsistent patterns of individual members. For each Delphi round, panelists were provided with results and the frequency distribution of responses recorded in the previous round, and were then invited to evaluate their previous answers and revise them if needed.

The analysis of voting results was performed by a non-voting methodologist (CR).

## Results

**Fig. 1:**
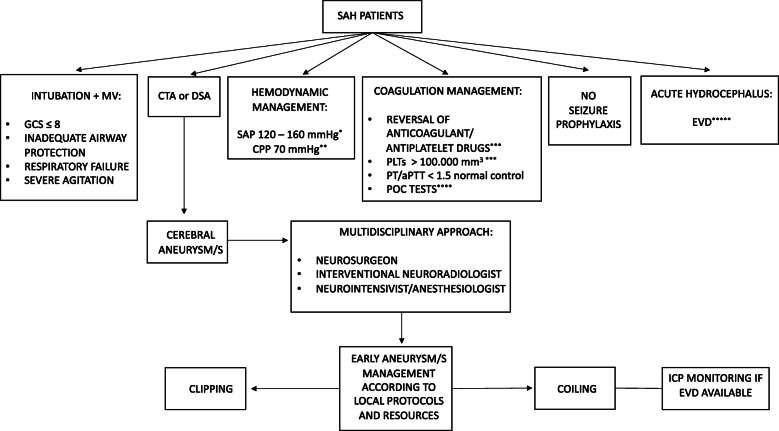
Consensus flow-chart. * according to patient’s clinical history (i.e., arterial hypertension, etc.) and/or radiological signs of intracranial hypertension. ** in case of ICP monitoring; for an accurate CPP estimation the arterial transducers need to be zeroed at the level of the tragus. *** SAH patients candidates for neurosurgical intervention. **** if available, to assess and optimize the coagulation function in salvageable SAH patients taking the NOACs and/or antiplatelet drugs. ***** before aneurysm treatment, in case of intracranial hypertension, drain small volumes of CSF to reduce the risk of rebleeding. *SAH* subarachnoid hemorrhage, *CTA* computed tomography angiography, *DSA* digital subtraction angiography, *POC* pint-of-care, *EVD* external ventricular drain, *ICP* intracranial pressure, *GCS* Glasgow coma scale, *CPP* cerebral perfusion pressure, *SAP* systolic arterial pressure, *PLTs* platelets, *PT* prothrombin time, *aPTT* activated partial thromboplastin time, *MV* mechanical ventilation

This consensus provided 19 statements (Table 1): 18 were strong recommendations, endorsed by more than 85% of participants, while we were unable to reach a consensus in one case. Figure 1 summarizes the recommendations in an operational flow chart. The consensus recommendations are listed below with the percentage of agreement.

**Table 1:** List of consensus recommendations

N.	Recommendation	Level
1	We recommend that all salvageable patients (i.e. patients who may recover, at least to some extent, with appropriate treatment) with a spontaneous SAH, according to local expertise and availability, must undergo a CTA or DSA.	***Strong recommendation***
2	We recommend that SAH patients in coma (GCS score ≤ 8) and/or with inadequate airway protection or respiratory failure need to be sedated, intubated and mechanically ventilated.	***Strong recommendation***
3	We recommend that SAH patients with severe agitation, despite mild sedation and pain control, need to be sedated, intubated and mechanically ventilated.	***Strong recommendation***
4	We recommend the maintenance of a PLTs count > 100.000/mm^3^ in all salvageable SAH patients candidates for neurosurgical intervention.	***Strong recommendation***
5	We recommend the reversal of antiplatelet drugs in all salvageable SAH patients, candidates for neurosurgical intervention.	***Strong recommendation***
6	We recommend the maintenance of a PT/aPTT value of < 1.5 normal control in all salvageable SAH patients.	***Strong recommendation***
7	We recommend the reversal of anticoagulant drugs in all salvageable SAH patients candidates for neurosurgical intervention.	***Strong recommendation***
8	We recommend, if available, the utilization of POC tests (e.g. TEG and ROTEM) to assess and optimize the coagulation function in salvageable SAH patients taking the NOACs and/or antiplatelets drugs.	***Strong recommendation***
9	We are unable to provide any recommendation regarding the routine administration of tranexamic acid for a short-term therapy (< 24 h from SAH) before aneurysm treatment to prevent rebleeding.	***No recommendation***
10	We recommend in all salvageable comatose SAH patients with acute hydrocephalus to rapidly undergo EVD placement before aneurysm management.	***Strong recommendation***
11	We recommend, before aneurysm treatment, for the management of intracranial hypertension related to acute hydrocephalus, the drainage (EVD available) of small volumes of CSF to reduce the risk of rebleeding.	***Strong recommendation***
12	We recommend, in case of EVD placement before aneurysm/s management, the ICP monitoring, during endovascular coiling.	***Strong recommendation***
13	We recommend the maintenance of a SAP between 120 and 160 mmHg to avoid aneurysmal rebleeding and to ensure an adequate CPP. Individualized arterial pressure targets considering patient’s clinical history (i.e., arterial hypertension) and/or radiological signs of intracranial hypertension seem reasonable.	***Strong recommendation***
14	We recommend the maintenance of SAP values close to the lower limit (120 mmHg) in patients without a history of arterial hypertension and/or radiological signs of elevated ICP.	***Strong recommendation***
15	We recommend the maintenance of SAP values close to the upper limit (160 mmHg), avoiding fluctuations, for patients with a history of arterial hypertension and/or radiological signs of elevated ICP.	***Strong recommendation***
16	We recommend, in case of ICP monitoring, the maintenance of a CPP of 70 mmHg*. * for an accurate CPP estimation the arterial transducers need to be zeroed at the level of the tragus.	***Strong recommendation***
17	We recommend against seizure prophylaxis in salvageable SAH patients without seizures (observed clinically and/or with EEG).	***Strong recommendation***
18	We recommend that ruptured cerebral aneurysm/s be secured early according to local protocols and resources.	***Strong recommendation***
19	We recommend a strict collaboration between the interventional neuroradiologist, the neurosurgeon, the neurointensivist/anesthesiologist to find the best strategy (clips or coils) to secure the ruptured cerebral aneurysm/s.	***Strong recommendation***

*SAH* subarachnoid hemorrhage, *CTA* computed tomography angiography, *DSA* digital subtraction angiography, *POC* pint-of-care, *TEG* thromboelastography, *ROTEM* rotational thromboelastometry, *NOACs* novel oral anticoagulants, *EVD* external ventricular drain, *CSF* cerebrospinal fluid, *EEG* electroencephalogram, *ICP* intracranial pressure, *GCS* Glasgow coma scale, *CPP* cerebral perfusion pressure, *SAP* systolic arterial pressure, *PLTs* platelets, *PT* prothrombin time, *aPTT* activated partial thromboplastin time

### Recommendation 1

We recommend that all salvageable patients (i.e., patients who may recover, at least to some extent, with appropriate treatment) with a spontaneous SAH, according to local expertise and availability, must undergo a computed tomography angiography (CTA) or digital subtraction angiography (DSA) (agreement 100%, strong recommendation).

### Recommendation 2

We recommend that SAH patients in coma [Glasgow Coma Scale (GCS) score ≤ 8] and/or with inadequate airway protection or respiratory failure need to be sedated, intubated and mechanically ventilated (agreement 95.5%, strong recommendation).

### Recommendation 3

We recommend that SAH patients with severe agitation, despite mild sedation and pain control, need to be sedated, intubated, and mechanically ventilated (agreement 86.5%, strong recommendation).

### Recommendation 4

We recommend the maintenance of a platelets (PLTs) count > 100.000/mm3 in all salvageable SAH patients candidates for neurosurgical intervention (agreement 100%, strong recommendation).

### Recommendation 5

We recommend the reversal of antiplatelet drugs in all salvageable SAH patients, candidates for neurosurgical intervention (agreement 86.5%, strong recommendation).

### Recommendation 6

We recommend the maintenance of a prothrombin time (PT)/ activated partial thromboplastin time (aPTT) value of < 1.5 normal control in all salvageable SAH patients (agreement 86.5%, strong recommendation).

### Recommendation 7

We recommend the reversal of anticoagulant drugs in all salvageable SAH patients candidates for neurosurgical intervention (agreement 100%, strong recommendation).

### Recommendation 8

We recommend, if available, the utilization of point-of-care (POC) tests [e.g., thromboelastography (TEG) and rotational thromboelastometry ROTEM] to assess and optimize the coagulation function in salvageable SAH patients taking the novel oral anticoagulants (NOACs) and/or antiplatelet drugs (agreement 91%, strong recommendation).

### Recommendation 9

We are unable to provide any recommendation regarding the routine administration of tranexamic acid for a short-term therapy (< 24 h from SAH) before aneurysm treatment to prevent rebleeding (agreement 58%, no recommendation).

### Recommendation 10

We recommend in all salvageable comatose SAH patients with acute hydrocephalus to rapidly undergo external ventricular drain (EVD) placement before aneurysm management (agreement 91%, strong recommendation).

### Recommendation 11

We recommend, before aneurysm treatment, for the management of intracranial hypertension related to acute hydrocephalus, the drainage (EVD available) of small volumes of cerebrospinal fluid (CSF) to reduce the risk of rebleeding (agreement 86%, strong recommendation).

### Recommendation 12

We recommend, in case of EVD placement before aneurysm/s exclusion, the intracranial pressure (ICP) monitoring, during endovascular coiling (agreement 91%, strong recommendation).

### Recommendation 13

We recommend the maintenance of a systolic arterial pressure (SAP) between 120 and 160 mmHg to avoid aneurysmal rebleeding and to ensure an adequate cerebral perfusion pressure (CPP). Individualized arterial pressure targets considering the patient’s clinical history (i.e., arterial hypertension) and/or radiological signs of intracranial hypertension seem reasonable (agreement 95.5%, strong recommendation).

### Recommendation 14

We recommend the maintenance of SAP values close to the lower limit (120 mmHg) in patients without a history of arterial hypertension and/or radiological signs of elevated ICP (agreement 95.5%, strong recommendation).

### Recommendation 15

We recommend the maintenance of SAP values close to the upper limit (160 mmHg), avoiding fluctuations, for patients with a history of arterial hypertension and/or radiological signs of elevated ICP (agreement 91%, strong recommendation).

### Recommendation 16

We recommend, in case of ICP monitoring, the maintenance of a CPP of 70 mmHg*

(agreement 86.5%, strong recommendation).

* For an accurate CPP estimation, the arterial transducers need to be zeroed at the level of the tragus.

### Recommendation 17

We recommend against seizure prophylaxis in salvageable SAH patients without seizures [observed clinically and/or with electroencephalogram (EEG)] (agreement 86.5%, strong recommendation).

### Recommendation 18

We recommend that ruptured cerebral aneurysm/s be secured early according to local protocols and resources (agreement 91%, strong recommendation).

### Recommendation 19

We recommend a strict collaboration between the interventional neuroradiologist, the neurosurgeon, the neurointensivist/anesthesiologist to find the best strategy (clips or coils) to secure the ruptured cerebral aneurysm/s (agreement 100%, strong recommendation).

## Discussion

Once admitted to a referral center, SAH patients may present a number of problems, either directly caused by the aneurysmal bleeding or depending on comorbidities, medications, etc. To assist in some simple, but crucial, decisions (who and when requires tracheal intubation, for instance), panelists have formulated a list of practical recommendations which may orient the treating team in various treatment steps, as illustrated in Fig. 1. These recommendations do not cover the full spectrum of airways, hemodynamic and coagulation problems; for aspects not peculiar of SAH, we refer to previous consensus and clinical recommendations of the SIAARTI [6]. Furthermore, a few items deserve to be discussed:

### Imaging before aneurysm management

Vessel imaging to identify the bleeding source is a key step in patients with SAH [7, 8, 11]. In this regard, DSA is considered the gold standard [7, 8, 11]. However, DSA is invasive, time-consuming, costly, and associated with neurologic complications [11, 12]. CTA, demonstrating a good sensitivity and specificity when compared to DSA [13], is increasingly utilized to detect cerebral aneurysm; in this regard, in many cases neurosurgeons can make the decision to proceed to clip based only on CTA [12], and recommend DSA only if CTA is inconclusive [7, 8]. Based on this, the panel agreed that the choice of performing CTA or DSA to identify the bleeding source and to choose the best treatment strategy should be made according to local availability and expertise.

### EVD and CSF drainage before aneurysm management

Acute hydrocephalus, occurring in up to 30% of SAH patients, can be associated with intracranial hypertension and reduced CPP [11]. In this situation, the placement of an EVD can be lifesaving [7, 8, 11]. However, excessive or rapid CSF drainage can be associated with an increase in the intra-aneurysmal transmural pressure precipitating rebleeding and need to be avoided [14, 15]. Considering the above, we recommend the placement of an EVD in all salvageable comatose SAH patients with acute hydrocephalus and the careful, progressive drainage of CSF to minimize the risk of rebleeding.

### ICP monitoring during endovascular coiling and CPP management

Episodes of elevated ICP and reduced CPP have been recently observed during endovascular coiling and could be associated with poor neurological outcomes [16]. Although more data are needed on this topic, we recommend ICP monitoring during endovascular coiling when an EVD has been inserted before aneurysm/s management.

CPP values < 70 mmHg have been associated with an increased risk of cerebral ischemia in poor-grade SAH patients [15, 17]. We therefore recommend the maintenance of a CPP of 70 mmHg. For an accurate CPP measurement, we suggest that the arterial transducer be zeroed at the level of the tragus according to recommendations for traumatic brain injury (TBI) patients [18].

### Seizure prophylaxis

Seizures can occur after SAH [11, 12, 15]. Recognized risk factors include thick subarachnoid clot, intracerebral hematoma, delayed infarction, and middle cerebral artery’s aneurysms [11, 12, 15]. Despite the association of seizures with poor neurological outcomes and rebleeding, there is a lack of consensus regarding the administration of prophylactic anticonvulsant therapy after SAH [7, 8]. In particular, phenytoin utilization has been associated not only with worse long-term cognitive outcomes [19] but also with reduced nimodipine plasma concentrations by induction of CYP450 [20]. Data regarding newer anticonvulsant drugs are needed. Based on the limited available evidence, we recommend against seizure prophylaxis in salvageable SAH patients without seizures (clinically observed and/or documented with the EEG).

### Aneurysm management

Aneurysm repair with surgical clipping or endovascular coiling is the only effective treatment to prevent rebleeding and should be performed as early as feasible [7, 8]. A recently published retrospective observational study on 575 aneurysmal SAH patients showed that more favorable outcomes (discharge at home and survival at 12 months) were achieved when surgical treatment occurred at approximately 12.5 h from SAH [21]. After the publication of the International Subarachnoid Aneurysm Trial (ISAT), comparing coils vs. clips utilization after SAH, the aneurysm treatment has mostly shifted from surgical clipping to endovascular coiling [11, 12, 15, 22, 23]. Patients in the coils group had a better neurological outcome 1 year after SAH and a lower risk of seizures compared to the clips group. Even after 10 years, patients in the endovascular coiling group had a better outcome in terms of probability of death and disability, despite a lower incidence of incomplete aneurysm occlusion and a lower risk of rebleeding in the surgical group [24]. The treatment of choice, however, has to be individualized and depends on multiple factors [11, 12, 15]. Clearly, this topic is outside the aims of this consensus. What has to be emphasized, however, is the importance of a multidisciplinary approach, where neurosurgeons, interventional neuroradiologists, and neurointensivists work together [7, 8, 11] and co-operate for an early aneurysm closure.

## Limitations

This consensus is not based on a systematic literature review, and this limitation has to be clearly acknowledged. We opted for a consensus based on clinical expertise, well aware that this approach has weaknesses [25].

## Conclusions

Our aim has been to provide practical suggestions on topics where the published evidence did not support strong statements. While deeper analyses of the literature are always welcome, we felt useful to indicate choices based on the panel’s expertise.

## Abbreviations

SAH: Subarachnoid hemorrhage
SIAARTI: Italian Society of Anesthesia and Intensive Care
CTA: Computed tomography angiography
DSA: Digital subtraction angiography
GCS: Glasgow coma scale
PLTs: Platelets
PT: Prothrombin time
aPTT: Activated partial thromboplastin time
POC: Point-of-care
TEG: Thromboelastometry
ROTEM: Rotational thromboelastography
NOACs: Novel oral anticoagulants
EVD: External ventricular drain
CSF: Cerebrospinal fluid
ICP: Intracranial pressure
CPP: Cerebral perfusion pressure
SAP: Systolic arterial pressure
EEG: Electroencephalogram
